# Differences in CD44 Surface Expression Levels and Function Discriminates IL-17 and IFN-γ Producing Helper T Cells

**DOI:** 10.1371/journal.pone.0132479

**Published:** 2015-07-14

**Authors:** Julia Schumann, Katarina Stanko, Ulrike Schliesser, Christine Appelt, Birgit Sawitzki

**Affiliations:** 1 Institute of Medical Immunology, Charité University Medicine, Berlin, Germany; 2 Berlin Brandenburg Center for Regenerative Therapies, Charité University Medicine, Berlin, Germany; University of Iowa, UNITED STATES

## Abstract

CD44 is a prominent activation marker which distinguishes memory and effector T cells from their naïve counterparts. It also plays a role in early T cell signaling events as it is bound to the lymphocyte-specific protein kinase and thereby enhances T cell receptor signalling. Here, we investigated whether IFN-γ and IL-17 producing T helper cells differ in their CD44 expression and their dependence of CD44 for differentiation. Stimulation of CD4^+^ T cells with allogeneic dendritic cells resulted in the formation of three distinguishable populations: CD44^+^, CD44^++^ and CD44^+++^. *In vitro* and *in vivo* generated allo-reactive IL-17 producing T helper cells were mainly CD44^+++^ as compared to IFN-γ^+^ T helper cells, which were CD44^++^. This effect was enhanced under polarizing conditions. T helper 17 polarization led to a shift towards the CD44^+++^ population, whereas T helper 1 polarization diminished this population. Furthermore, blocking CD44 decreased IL-17 secretion, while IFN-γ was barely affected. Titration experiments revealed that low T cell receptor and CD28 stimulation supported T helper 17 rather than T helper 1 development. Under these conditions CD44 could act as a co-stimulatory molecule and replace CD28. Indeed, rested CD44^+++^CD4^+^ T cells contained already more total and especially phosphorylated zeta-chain-associated protein kinase 70 as compared to CD44^++^ cells. Our results support the notion, that CD44 enhances T cell receptor signaling strength by delivering lymphocyte-specific protein kinase, which is required for induction of IL-17 producing T helper cells.

## Introduction

CD44 is a type I transmembrane glycoprotein and expressed by many different cell types. Although it is encoded only by a single gene, cells can express multiple CD44 variants, due to alternative splicing and posttranslational modification [1, 2]. CD44 has been described to bind several ligands (e.g. fibronectin [3], osteopontin [4], collagen [5]) but the most known one is hyaluronan.

T cells express the minimal so called standard version of CD44, which is the product of ten exons [1]. CD44 is one of the most commonly used activation markers for T cells. After antigen encounter, T cells rapidly up-regulate CD44 and its expression is also maintained in memory T cells [6]. Besides its usage as an activation and memory marker, CD44 mediates several other functions, which can be attributed to three different properties [1]. CD44 can interact with components of the extracellular matrix and rolling of lymphocytes by the interaction of CD44 and hyaluronan was one of the first functions ascribed to this protein [7]. Additionally, CD44 has also been described to interact with the cytoskeleton [8, 9] and to function as a co-receptor in T cell activation [10].

No intrinsic enzymatic activity is described for the intracellular C-terminal part of CD44, but numerous publications showed, that it interacts with receptor tyrosine kinases, such as lymphocyte-specific kinase (LCK) and Fyn [11–14]. Nevertheless, it has not been observed, that binding of hyaluronan causes a conformational shift of the intracellular part. Supporting this, the extent of LCK-binding and phosphorylation seemed to be independent from CD44-crosslinking. However, crosslinking of CD44 led to activation of extracellular-signal regulated kinase and supported T cell stimulation [12]. Thus by recruiting LCK to active signalling sites, CD44 increased its availability and density [12]. Supporting this, a small amount of CD44 is located in lipid rafts and only there it is associated with LCK [13]. Some studies reported, that CD3-crosslinking led to a fusion of lipid rafts [15], which would increase the density of CD44 and LCK.

T helper (Th) cells play an essential role in the function and activation of the adaptive immune system. The dichotomy of Th1 and Th2 cells was originally defined by Mosmann *et al* [16]. To date several other Th cell subpopulations have been defined according to their ability to secrete cytokines, express master regulators, their role in defending pathogens and association with autoimmune diseases [17, 18]. IL-17 and IFN-γ are the hallmark cytokines of Th17 and Th1 cells, respectively [16, 19, 20].

Although no splice variants could be detected, which distinguish different Th cell subpopulations [21], several studies found a Th cell-specific role for CD44. It has been shown that in delayed-type hypersensitivity reactions the knock-out (KO) of *Cd44* reduced Th1 but enhanced Th2 cell responses [22]. Moreover, *in vitro* polarized CD4^+^ T cells from *Cd44*-KO mice showed reduced IL-17 and IFN-γ secretion, while IL-4 was enhanced. As a consequence *Cd44*-KO mice developed a milder experimental autoimmune encephalomyelitis (EAE) [23]. In addition, it could be shown, that CD44 enhanced the survival of memory Th1 cells since *Cd44*-KO mice could not build memory in an influenza model [21]. These studies suggest, that CD44 is rather important for Th1 and Th17-differentiation. But in contrast, in an airway hyper-responsiveness model *Cd44*-KO mice showed diminished Th2-mediated responses [24]. However, there are also studies, which illustrate a suppressive role for CD44. One of these studies described that *Cd44*-KO led to an increase in IL-17 and severe EAE, which is in contrast to the finding of Guan *et al*. [23, 25]. Taken together this suggests, that CD44 seems to be generally necessary for Th cell responses.

In contrast to studies on *Cd44*-KO mice our reported findings demonstrate, that the level of CD44 expression per cell distinguishes Th cell subpopulations. We found that allo-reactive IL-17 secreting CD4^+^ T cells have a higher surface CD44 expression as compared to their IFN-γ producing counterparts. This finding was true for *in vitro* and *in vivo* developing IL-17^+^CD4^+^ and IFN-γ^+^CD4^+^ T cells. Moreover, polarizing conditions strengthened this phenotype and differentiation of IL-17^+^CD4^+^ T cells was dependent on CD44 function. We could also confirm that Th17 cells preferentially develop under low-dose αCD3-treatment and low CD28 stimulation [26, 27]. Under exactly these conditions CD44 could strengthen the intracellular signal cascade and therefore serve as co-stimulatory molecule.

## Methods

### Mice

Adult (8–12 weeks) male C57BL/6 and BALB/c mice were purchased from Charles River Laboratories (Sulzfeld, Germany). All experiments were approved by the local review board and are in accordance with the guidelines of the Federation of European Laboratory Animal Science Associations (FELASA).

### Cell culture

To generate bone marrow-derived dendritic cells (BMDCs) tibiae and femora of BALB/c mice were flushed. Single cell suspension was prepared and erythrocytes were lysed. Precursor cells were cultured for eight days in presence of 6 ng/ml GM-CSF (Miltenyi Biotec, Bergisch Gladbach, Germany). Media was exchanged every two days. For allogeneic co-cultures single cell suspensions from splenocytes and lymph node cells were prepared. Cells were enriched for CD4^+^ T cells using CD4^+^ T cell isolation kit II (Miltenyi Biotec). If indicated cells were sorted for naïve cells (FACS-sorting: CD4^+^CD45RB^high^, MACS-sorting: untouched naïve CD4+ T cell kit, Miltenyi Biotec) or labelled with the proliferation dye eFluor450 (eBioscience, San Diego, USA) according to the manufacturer`s instructions. 2.5 × 10^6^ CD4^+^ T cells and 2 × 10^5^ allogeneic BMDCs per 48-well were co-cultured for four days in RPMI containing 50 μM β-mercaptoethanol, 10% FCS, 100 U/ml penicillin and 100 μg/ml streptomycin (all Biochrom AG, Berlin, Germany) at 37°C and 5% CO_2_. Analysis of the polarized cultures was performed on day three or four as indicated.

For polyclonal cell culture experiments 1.2 × 10^6^ CD4^+^ T cells and 1.2 × 10^6^ splenocytes per 48-well were cultured for four days in supplemented RPMI at 37°C and 5% CO_2_. 0.01 or 1 μg/ml αCD3 (clone: 145-2C11, eBioscience) was added.

For polyclonally stimulated cultures under T helper cell polarizing conditions cells were FACS-sorted for naïve T cells (CD3^+^CD4^+^CD45RB^high^). Cells, which did not express CD3, were used as APCs. Polyclonal stimulation in polarization experiments was done with 3 μg/ml plate-bound αCD3 and 1 μg/ml αCD28 (clone: 37.51, eBioscience). Cells were seeded in a T cell to APC ratio of 1:5. The composition of the polarization cocktails is described below. 48 h after stimulation cells were harvested and seeded in new wells without adding new media. Four days later intracellular cytokine staining was performed.

### Cell sorting

Cell sorting was done on a FACSAria II cell sorter (BD Biosciences, Heidelberg, Germany).

### Treatments

Addition of αCD44 or CTLA-4-Ig is specified in the figures (both BD Biosciences). In experiments where αCD44 was added, results are displayed as ratio of cytokine production in αCD44-treated and untreated cultures. For Th1 cell polarization 10 ng/ml murine recombinant IL-12 (Peprotech, Hamburg, Germany) and 10 μg/ml αIL-4 (clone: 11-B11, DRFZ, Berlin Germany) was added. For Th17 cell polarization 20 ng/ml murine recombinant IL-6 (Peprotech), 10 ng/ml murine recombinant IL-23 (Peprotech), 1 ng/ml human recombinant TGF-β, 10 μg/ml αIL-4 and αIFN-γ (clone: R4-6A2 and XMG1.2, BioLegend, San Diego, USA) was added.

### Flow cytometry

For intracellular cytokine staining cells were stimulated with 1 μg/ml ionomycin (Biotrend chemicals, cologne, Germany) and 10 ng/ml phorbol 12-myristate 13-acetate (PMA) (Sigma-Aldrich, St. Louis, USA) for four hours. For the last two hours 2 μg/ml brefeldin A (Sigma-Aldrich) was added. Cells were washed and re-suspended in staining buffer (PBS supplemented with 2% FCS and 0.01% sodium azide) and stained with Fc-Block (24G2, BD Bioscience). In order to discriminate living and dead cells, a specific staining was included. This allows us to determine the percentage of living cells within on total single cells. Live/dead cell staining was performed with a 1:10 dilution of the fixable dead cell staining dye eFluor506 (eBioscience) according to manufacturer’s instructions. After washing the cells, surface staining was performed. Monoclonal antibodies coupled to different fluorescent dyes against the following antigens were used: CD3 (17A2, BioLegend), CD4 (RM4-5, BioLegend), CD44 (IM7, BioLegend or KM201, Abcam) and CD45RB (C363-16A, BioLegend). Intracellular cytokine staining was performed using Cytofix/Cytoperm buffer (BD Biosciences) and Perm/Wash buffer (BioLegend) according to manufacturer`s protocol. Monoclonal antibodies coupled to different fluorescent dyes against the following antigens were used: IFN-γ (XMG1.2), TNF-α (MP6-XT22) (both BioLegend) and IL-17 (clone ebio17E7, eBioscience). Cells were measured on a LSR II or LSRFortessa flow cytometer (BD Biosciences). Data analysis was performed using FlowJo software v9 (Treestar, Ashland, USA). Dead cells and doublets were excluded from analysis (S1 File). To avoid contamination of CD4^+^ T cells with BMDCs in allogeneic co-cultures cells were pre-gated on CD3^+^ or CD4^+^ cells.

As described cells were stained with a proliferation dye before co-culturing. Tracking cell populations is possible due to its progressive reduction after cell division. Calculation of cell division and estimation of the resulting cell generations was done applying FlowJo software. The proliferation platform of this software draws gates in order to separate each generation. Afterwards, the different generations were analyzed with regard to geometric mean fluorescence (gMFI) of CD44 and cytokine production.

### Phospho-zeta-chain-associated kinase 70 (ZAP-70) and ZAP-70 staining

CD4^+^ T cells were harvested on day four after stimulation with allogeneic BMDCs. Since most of the BMDCs were adherent CD4^+^ T cells could be separated from them by transferring them into a new well with fresh medium. 24 h after resting (no additional stimulation by BMDCs) total ZAP-70 and ZAP-70 phosphorylation was measured using αZAP-70 (1E7.2, BioLegend), αZAP-70 (Y319)/Syk (Y352) (clone: 17A/P-ZAP70, BD Bioscience), Cytofix Buffer and Phosflow Perm Buffer III (both BD Biosciences) according to manufacturer`s instructions (BD phosflow protocoll III). CD44 staining was performed before fixation. If CD44 was blocked with αCD44 clone IM7, staining was performed using αCD44 clone KM201 (Abcam, Cambridge, England).

### Skin transplantation (sTx)

C57BL/6 mice received a transplant (1.1 x 0.8 cm) from BALB/c full-thickness tail skin. After transplantation weight changes and signs of skin rejection were documented. Mice were sacrificed on days 7, 14 and 28 post transplantation. Cells from spleen and lymph nodes were isolated and re-stimulated with 10 ng/ml PMA and 1 μg/ml ionomycin for four hours. Brefeldin A (2 μg/ml) was added after the first two hours. Afterwards, surface and intracellular cytokine staining was performed.

### Statistical analysis

Paired T-test or Wilcoxon rank sum test was used for the statistical analysis to compare groups of different treatments, different cell types of the same animal or co-cultures. Mann-Whitney test was used to compare unpaired groups. Friedman test and *post-hoc* Dunn`s comparison were used to compare paired values of more than two groups. Kruskal-Wallis test and *post-hoc* Dunn`s comparison were used to compare non-paired values of more than two groups. Two-way ANOVA and post-hoc Sidak’s multiple comparison test were used to compare two groups and more than one factor. P-Values ≤ 0.05 are considered as significant. Statistical analyses were done using GraphPad Prism v6 (GraphPad Software Inc., La Jolla, USA).

## Results

### Level of CD44 expression distinguishes allo-reactive IL-17 from IFN-γ producing CD4^+^ T cells *in vitro* and *in vivo*

**Fig 1:**
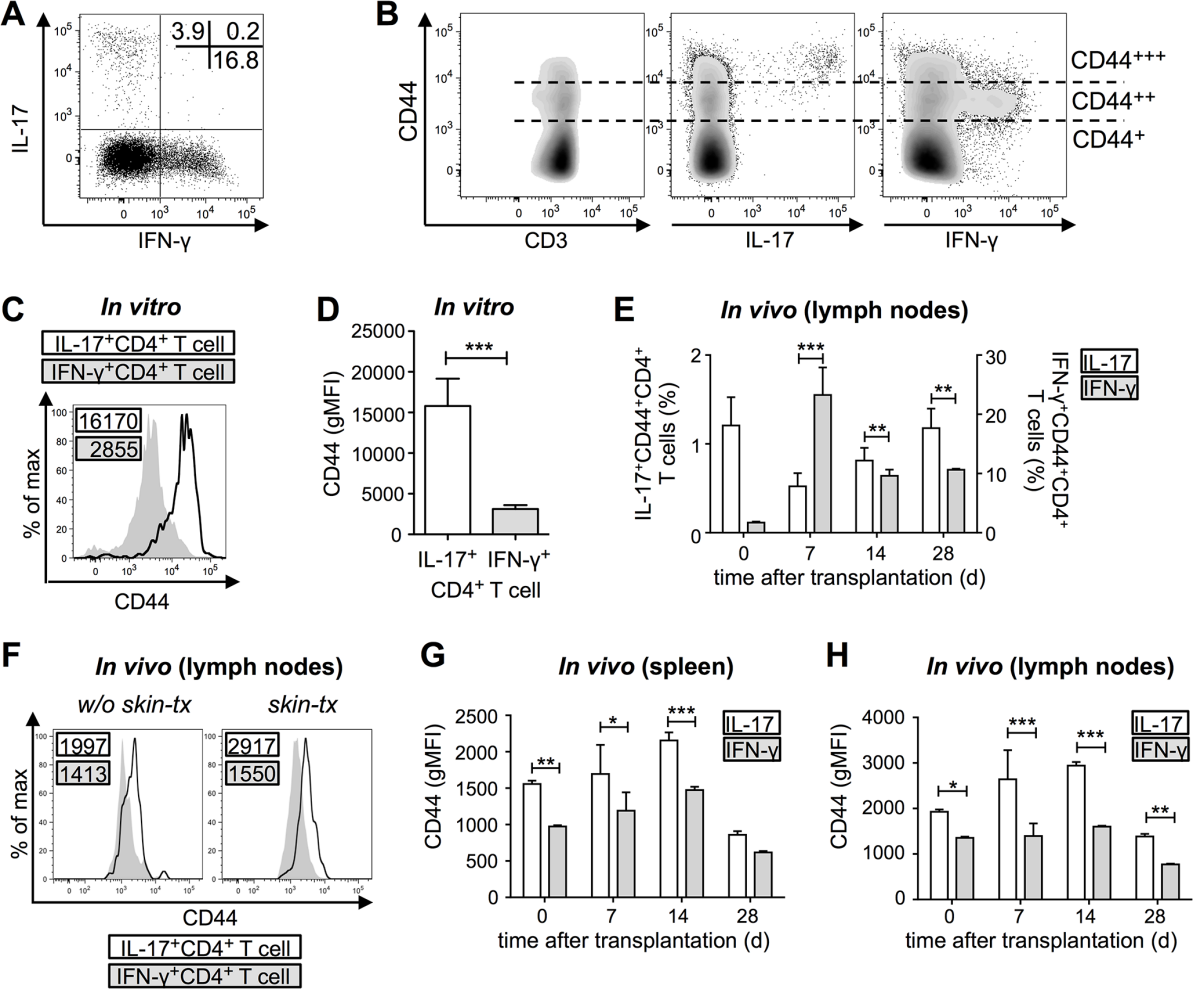
CD44 expression level discriminates allo-reactive IL-17^+^ and IFN-γ^+^ CD4^+^ T cells. (A) Cytokine production in CD4^**+**^ T cells four days after allogeneic co-culture with bone-marrow derived dendritic cells. Percentage of cells is indicated in the dot plot. (B) CD44 subpopulations of CD4^**+**^ T cells and cytokine production within these subpopulations four days after allogeneic co-culture. (C) CD44 expression level of *in vitro* generated IFN-γ^**+**^CD4^**+**^ (grey) and CD4^**+**^IL17^**+**^ T cells (black line). Values for the geometric mean fluorescence intensity (gMFI) for CD44 are indicated in the histogram. (D) CD44 expression level (gMFI) of *in vitro* generated IL-17^**+**^CD4^**+**^ and IFN-γ^**+**^CD4^**+**^ T cells (n = 13, *M* ± SEM, Wilcoxon rank sum test). (E) IL-17 and IFN-γ production prior to as well as seven, 14 and 28 days after allogeneic skin transplantation (n = 3–4, *M* ± SEM, 2-way ANOVA and *post-hoc* Sidak`s multiple comparison). (F) CD44 expression level of *in vivo* formed IFN-γ^**+**^CD4^**+**^ (grey) and CD4^**+**^IL17^**+**^ T cells (black line) from lymph nodes after transplantation (skin-tx) and without transplantation (w/o). GMFI for CD44 is indicated in the histograms. (G) CD44 expression level (gMFI) of *in vivo* formed IL-17^**+**^CD4^**+**^ and IFN-γ^**+**^CD4^**+**^ T cells from spleen prior to as well as seven, 14 and 28 days after transplantation (n = 3–4, *M* ± SEM, 2-way ANOVA and *post-hoc* Sidak’s multiple comparison. (H) CD44 expression level (gMFI) of *in vivo* formed IL-17^**+**^CD4^**+**^ and IFN-γ^**+**^CD4^**+**^ T cells from lymph nodes after transplantation (n = 3–4 *M* ± SEM, 2-way ANOVA and *post-hoc* Sidak’s multiple comparison). Statistical significance (*p*) is within the groups is indicated in the figures (*p ≤ 0.05, **p ≤ 0.01, ***p ≤ 0.001).

Magnetically CD4^+^-sorted T cells were stimulated with allogeneic bone marrow-derived dendritic cells (BMDCs) for four days. Intracellular cytokine staining was done in order to detect levels of IFN-γ and IL-17 within CD4^+^ T cells. Allogeneic co-culture led to the generation of IFN-γ^+^ and IL-17^+^CD4^+^ T cells (Fig 1A). Cells from this culture could be divided into three different subpopulations according to their CD44 expression: CD44^+^, CD44^++^ and CD44^+++^ (Fig 1B). Interestingly, *in vitro* generated allo-reactive IL-17-secreting CD4^+^ T cells showed a higher CD44 expression as compared to IFN-γ-secreting CD4^+^ T cells (Fig 1B and 1C). This effect was highly reproducible and therefore strongly significant (Fig 1D). To exclude that this observation was due to *in vitro* culture conditions we examined the CD44 surface level of *in vivo* generated allo-reactive IL-17^+^ and IFN-γ^+^CD4^+^ T cells. Therefore, C57BL/6 animals received a skin transplant from BALB/c mice. Prior to as well as seven, 14 and 28 days after transplantation cells from lymph nodes and spleen were isolated. Cells were re-stimulated with PMA and ionomycin in order to determine cytokine levels. As shown in Fig 1E, IFN-γ production peaked on day seven after transplantation and declined until day 28. In contrast, IL-17 production was not affected by the transplantation. Comparing IL-17^+^ and IFN-γ^+^CD4^+^ T cells from animals 14 days after transplantation, we also found a difference in the CD44 surface expression *in vivo* (Fig 1F). Interestingly, also IL-17^+^ cells of naïve mice, which did not receive a transplant, showed higher CD44 expression levels as compared to IFN-γ^+^ (Fig 1G and 1H). This was observed for each time point studied and true for spleen and lymph nodes (Fig 1G and 1H).

### Polarization strengthens differences in CD44 expression

**Fig 2:**
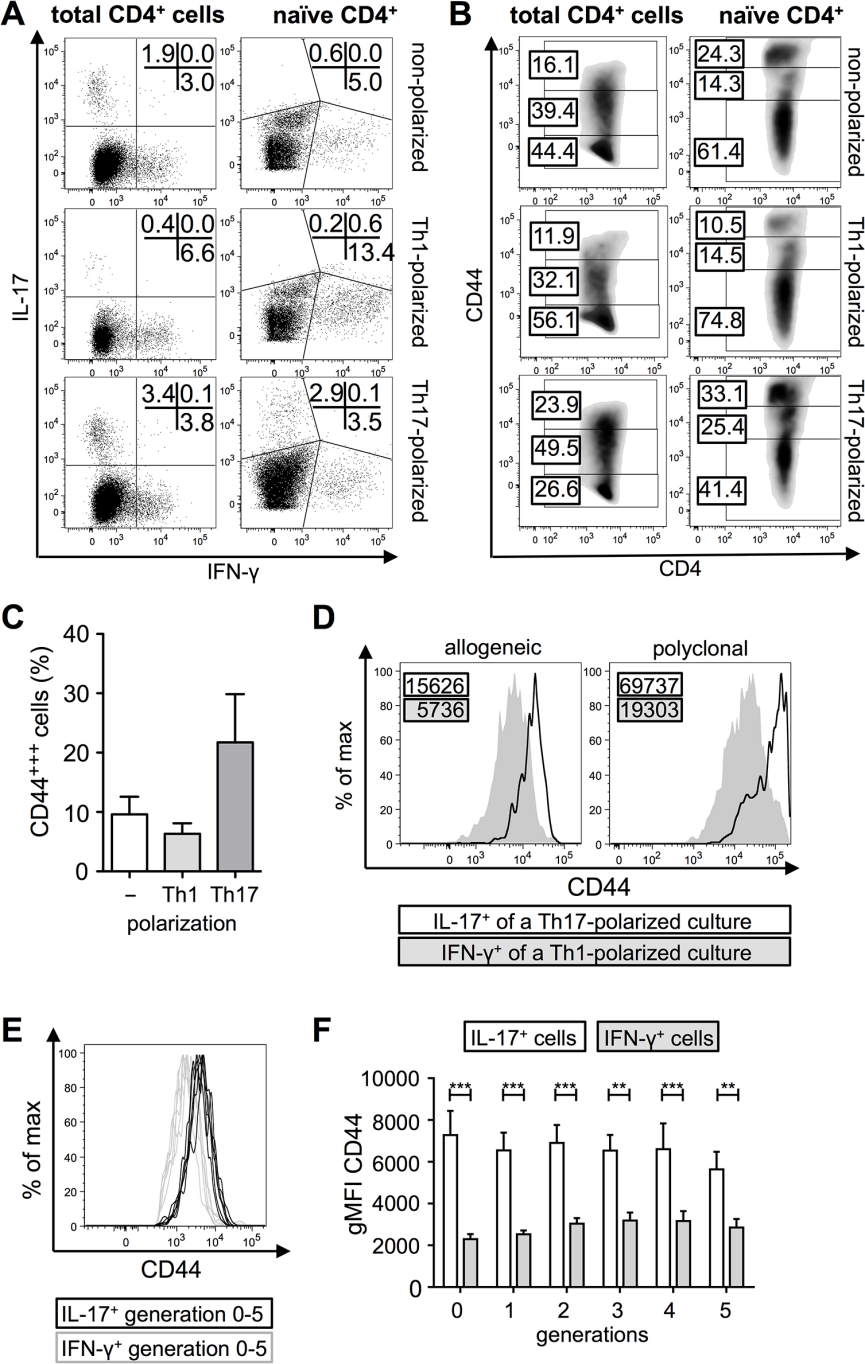
Differences in the CD44 expression level is strengthened by polarization. (A) Representative dot plot of IL-17 and IFN-γ production by CD4^**+**^ T cells in non-polarized, Th1- and Th17-polarized allogeneic co-cultures for either total CD4^**+**^ T cells or for naïve-sorted (CD45RB^**high**^) CD4^**+**^ T cells. Percentage of cells is indicated in the plot. Analysis was performed on day four after co-culture. (B) Representative dot plot of the CD44 surface level expression of CD4^**+**^ T cells in non-polarized, Th1- and Th17-polarized allogeneic co-cultures for either total CD4^**+**^ T cells or for naïve-sorted (CD45RB^**high**^) CD4^**+**^ T cells. Percentage of cells within different subpopulations is indicated in the plot. Analysis of total CD4^**+**^ T cells was performed on day four and of naïve-sorted on day three after co-culture. (C) Percentage of CD44^**+++**^CD4^**+**^ T cells (total CD4^**+**^ T cells) under different polarization conditions (n = 6–9, *M* ± SEM, Kruskal-Wallis test and *post-hoc* Dunn’s comparison, not significant). (D) Histogram overlay of geometric mean fluorescence intensity (gMFI) for CD44 of IFN-γ producers of Th1-polarized co-cultures (grey) and IL-17 producers of Th17-polarized co-cultures (black line). (E and F) GMFI for IFN-γ and IL-17 producers of different generations is shown as histogram overlay (E) and bar chart (F; n = 6, *M* ± SEM; 2-way ANOVA and *post-hoc* Sidak`s multiple comparison, **p ≤ 0.01, ***p ≤ 0.001).

If a higher CD44 expression would be a signature of IL-17 producing CD4^+^ T cells we hypothesized, that polarizing conditions will increase the observed differences in CD44 surface levels. Under allogeneic co-culture conditions Th cell polarization did not result in significantly higher frequencies of Th17 cells. This was most probably due to a high number of IFN-γ-secreting CD4^+^ T cells despite IFN-γ neutralization (Fig 2A). However, Th1-polarization indeed led to a reduction of the CD44^+++^ fraction, whereas Th17-polarization increased the CD44^+++^ population (Fig 2B and 2C). Also under polarizing conditions all IL-17-producing cells were contained within the CD44^+++^ fraction, whereas IFN-γ producing cells were CD44^++^ (data not shown). These results show that there is a strong association between high CD44 expression and the IL-17 production. To ensure that this effect was not due to previously formed memory T cells in the culture, we performed additional experiments with CD4^+^CD45RB^high^-sorted naïve T cells. Also with naïve-sorted CD4^+^ T cells the CD44^+++^ population increased upon Th17 cell polarization (Fig 2A and 2B). Moreover, IFN-γ^+^ cells from naïve-sorted Th1-polarized allogeneic co-cultures had a reduced CD44 surface expression as compared to IL-17^+^ cells (Fig 2D). Importantly, this was also true for polyclonally stimulated CD4^+^ T cells (Fig 2D).

In order to reveal, whether the high CD44 expression is due to a progression in cell divisions, we labelled cells with a proliferation dye before allogeneic stimulation. Thereby different cell generations were distinguishable and simultaneous measurement of IL-17 production and CD44 expression is possible for the individual cell generations. We found that differences between IFN-γ and IL-17 producing CD4^+^ T cells in the CD44 level is maintained for at least five generations after cell division. This is shown exemplarily in Fig 2E as an overlay for the first five generations of IL-17^+^ and IFN-γ^+^ CD4^+^ T cells and summarized in Fig 2F. Similar results were obtained for naïve-sorted CD4^+^ T cells (S2 File).

**Fig 3:**
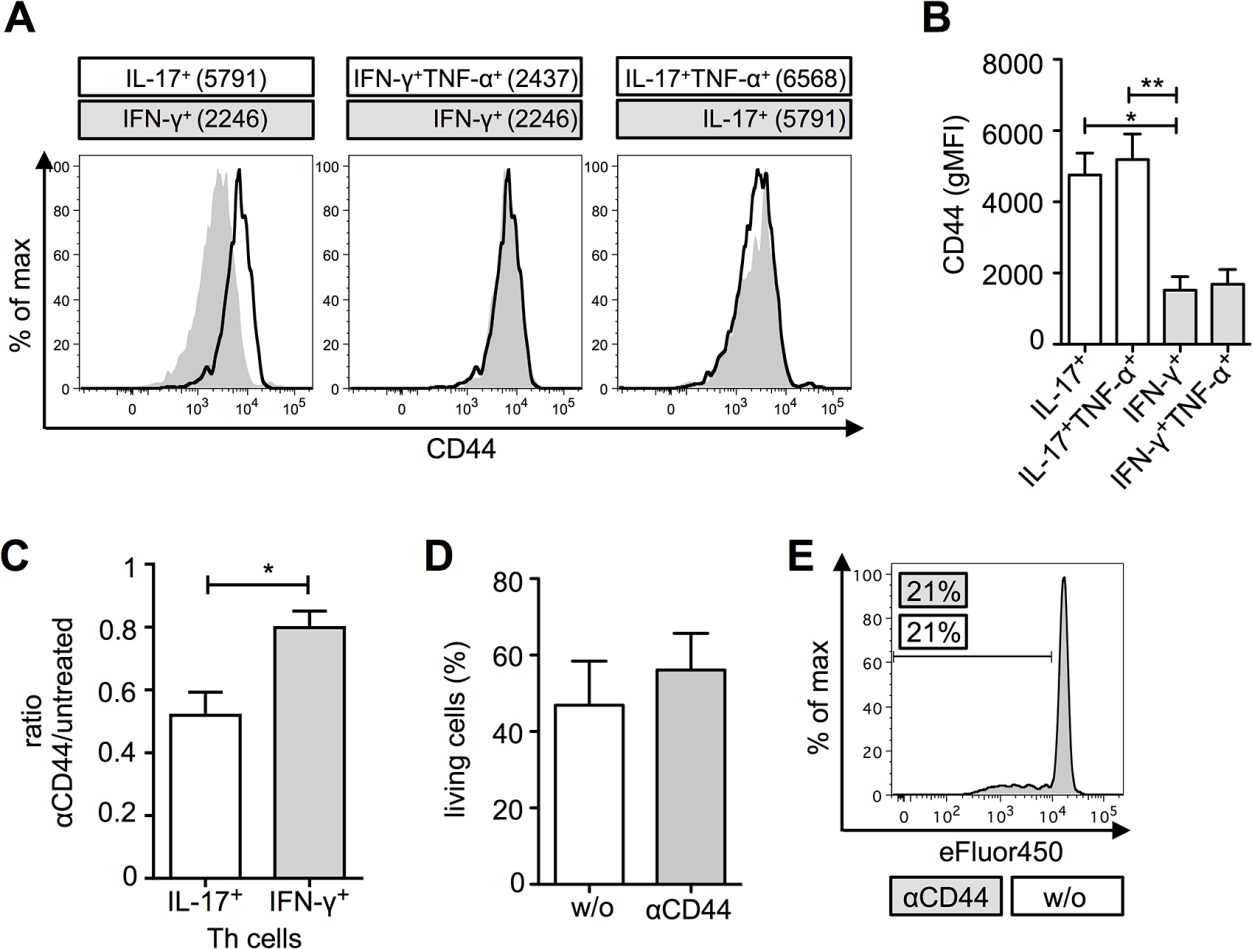
CD44 levels are independent of TNF-α expression and influences differentiation of IL-17^+^ cells. (A) Overlay of IFN-γ^**+**^ (grey) and IL-17^**+**^ (black), IFN-γ^**+**^ (grey) and IFN-γ^**+**^TNF-α^**+**^ (black), IL-17^**+**^ (grey) and IL-17^**+**^TNF-α^**+**^ (black) CD4^**+**^ T cells regarding their CD44 expression levels. Geometric mean fluorescence intensity (gMFI) for CD44 is indicated. (B) GMFI of CD44 for IL-17 and IFN-γ single producers, as well as for IL-17/TNF-α and IFN-γ/TNF-α double producing CD4^**+**^ T cells (n = 5, *M* ± SEM, Friedman test and post-hoc Dunn`s comparison). (C) Ratio of IL-17^**+**^ or IFN-γ^**+**^CD4^**+**^ T cells, respectively, in αCD44-treated versus untreated co-cultures (n = 5; *M* ± SEM; Wilcoxon rank sum test). (D) Living cells in allogeneic co-cultures treated with or without (w/o) αCD44 (n = 5; *M* ± SEM; Wilcoxon rank sum test, not significant). (E) Proliferation of CD4^**+**^ T cells in allogeneic co-cultures treated with (grey) or without (w/o, black line) αCD44. Statistical significance (*p*) is within the groups is indicated in the figures (*p ≤ 0.05, **p ≤ 0.01, ***p ≤ 0.001).

Moreover, the difference in the CD44 expression level seemed to be specific for Th1 and Th17 cells. Supporting this hypothesis, we also found that TNF-α, a cytokine, which can be produced by Th17 and Th1 cells, neither influenced the CD44 level of IL-17^+^ nor IFN-γ^+^CD4^+^ T cells. As a result IL-17/TNF-α double positive CD4^+^ T cells showed a similar CD44 level as IL-17 single positive cells (Fig 3A). The same was true for IFN-γ single positive and IFN-γ/TNF-α double positive CD4^+^ T cells (Fig 3A and 3B).

### Blockade of CD44 decreases IL-17 but not IFN-γ secretion

If a high CD44 expression would be necessary for IL-17 production of CD4^+^ T cells we speculated, that CD44 blockade by CD44-neutralizing antibodies will decrease the frequency of IL-17^+^CD4^+^ T cells in an allogeneic co-culture. When we added a neutralizing αCD44 antibody to our co-cultures we detected a significant reduction in IL-17^+^CD4^+^ T cells as compared to untreated co-cultures. Moreover, IFN-γ producing CD4^+^ T cells were only marginally affected as compared to IL-17^+^ cells (Fig 3C). This effect was not due to an increased cell death (Fig 3D) or an increased proliferation (Fig 3E) after treatment with αCD44. This supported our hypothesis, that a high CD44 expression is important for IL-17 development but obviously it is not essential. Thus, CD44 expression seemed to be more important for Th17 cells as compared to Th1 cells.

### CD44^+++^CD4^+^ T cells have a higher cellular activation

**Fig 4:**
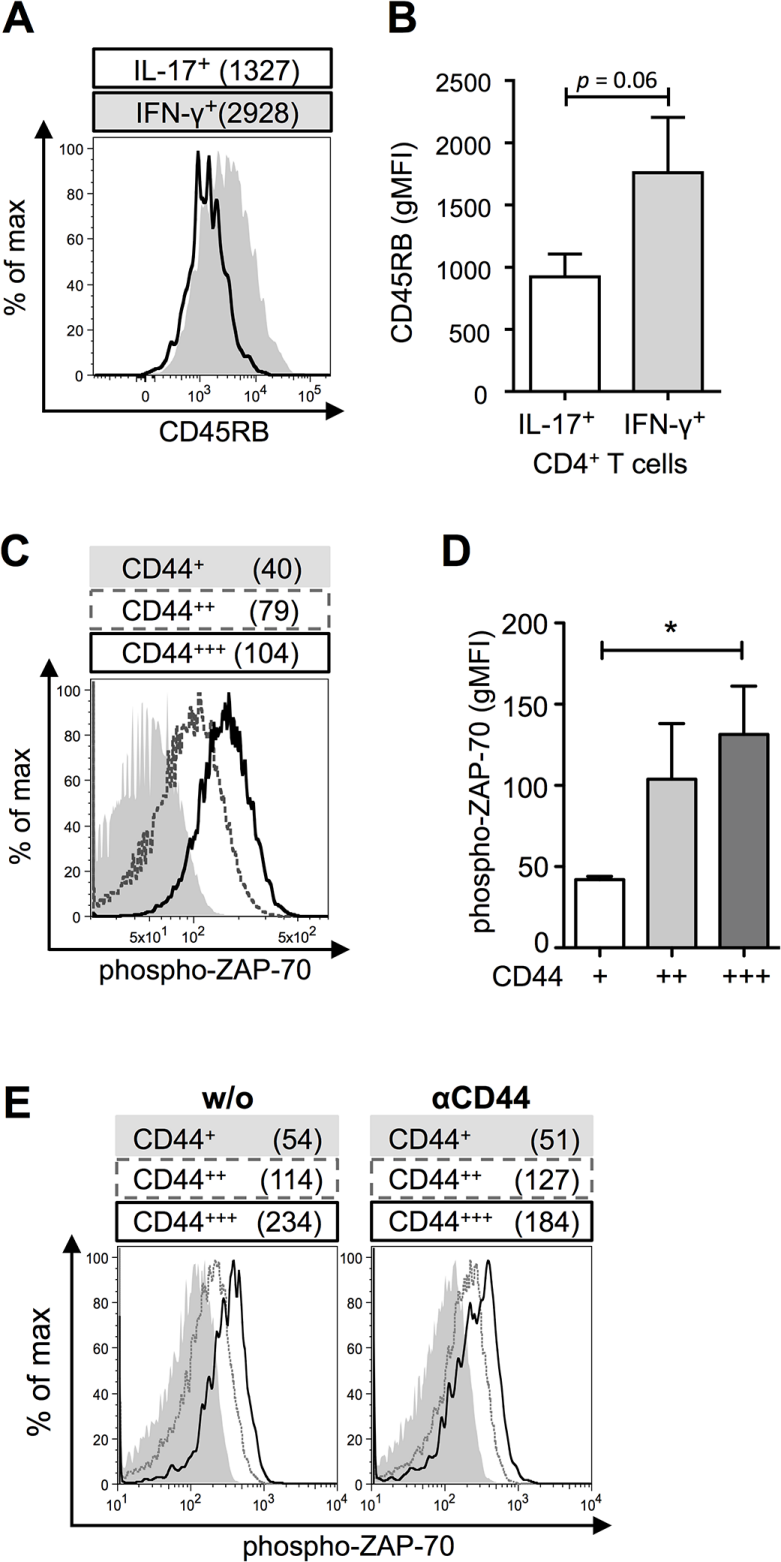
CD44^+++^CD4^+^ T cells have a high cellular activation status. (A) CD45RB expression level of *in vitro* generated IFN-γ^**+**^CD4^**+**^ (grey) and CD4^**+**^IL-17^**+**^ (black line) T cells. GMFI for CD45RB is indicated. (B) CD45RB geometric mean fluorescence intensity (gMFI) of IL-17^**+**^CD4^**+**^ and IFN-γ^**+**^CD4^**+**^ T cells (n = 5, *M* ± SEM, Wilcoxon rank sum test, *p* = 0.06). (C and D) Representative histogram (C) and summary (D) of the ZAP-70 phosphorylation for CD4^**+**^CD44^**+**^ (grey solid), CD44^**++**^ (grey dotted) and CD44^**+++**^ (black line) T cell populations. After four days of allogeneic co-culture cells have been rested for one day. Values for the gMFI of phospho-ZAP-70 are indicated. (E) GMFI of phospho-ZAP-70 of CD4^**+**^CD44^**+**^ (grey solid), CD44^**++**^ (grey dotted) and CD44^**+++**^ T cells (black line) with or without (w/o) αCD44 treatment. A representative histogram overlay out of four independent experiments is shown.

A higher CD44 expression might be a sign for an increased cellular activation status. Upon T cell activation CD44 expression is up-regulated but expression of other surface markers such as CD45RB is down-regulated [28]. In naïve and resting T cells CD45 is a positive regulator of T cell activation by dephosphorylating inhibitory residues. But during cell activation it can act as inhibitory signal by dephosphorylating activating residues in LCK. In the next step, we wanted to elucidate, whether IL-17^+^CD4^+^ T cells also differ from IFN-γ^+^CD4^+^ T cells in the expression of other surface marker such as CD45RB. Indeed, IL-17^+^CD4^+^ T cells showed a reduced expression of CD45RB as compared to IFN-γ^+^CD4^+^ T cells (Fig 4A and 4B).

Several publications described that the co-stimulatory properties of CD44 were due to its association with LCK [11–13]. Therefore, a higher CD44 expression might result in a higher density of LCK at the immunological synapse and thereby enhance the signalling. To address this question we measured the basal ZAP-70 phosphorylation following a one-day rest after allogeneic co-culture. We could measure a phosphorylation of ZAP-70 in all three CD44 subpopulations as compared to unstained fluorescence-minus-one controls (Figs A and B in S3 File). We found that cells with the highest CD44 level also displayed the highest amount of basal phosphorylated ZAP-70 as compared to CD44^+^ and CD44^++^CD4^+^ T cells (Fig 4C and 4D). Importantly, αCD44 treatment affected only the ZAP-70 phosphorylation of the CD44^+++^ population (Fig 4E, Fig C in S3 File). Interestingly, also total ZAP-70 levels increased with the amount of surface CD44 expression (Figs A and B in S4 File).

### Low T cell receptor (TCR) and CD28 stimulation supports Th17 development

**Fig 5:**
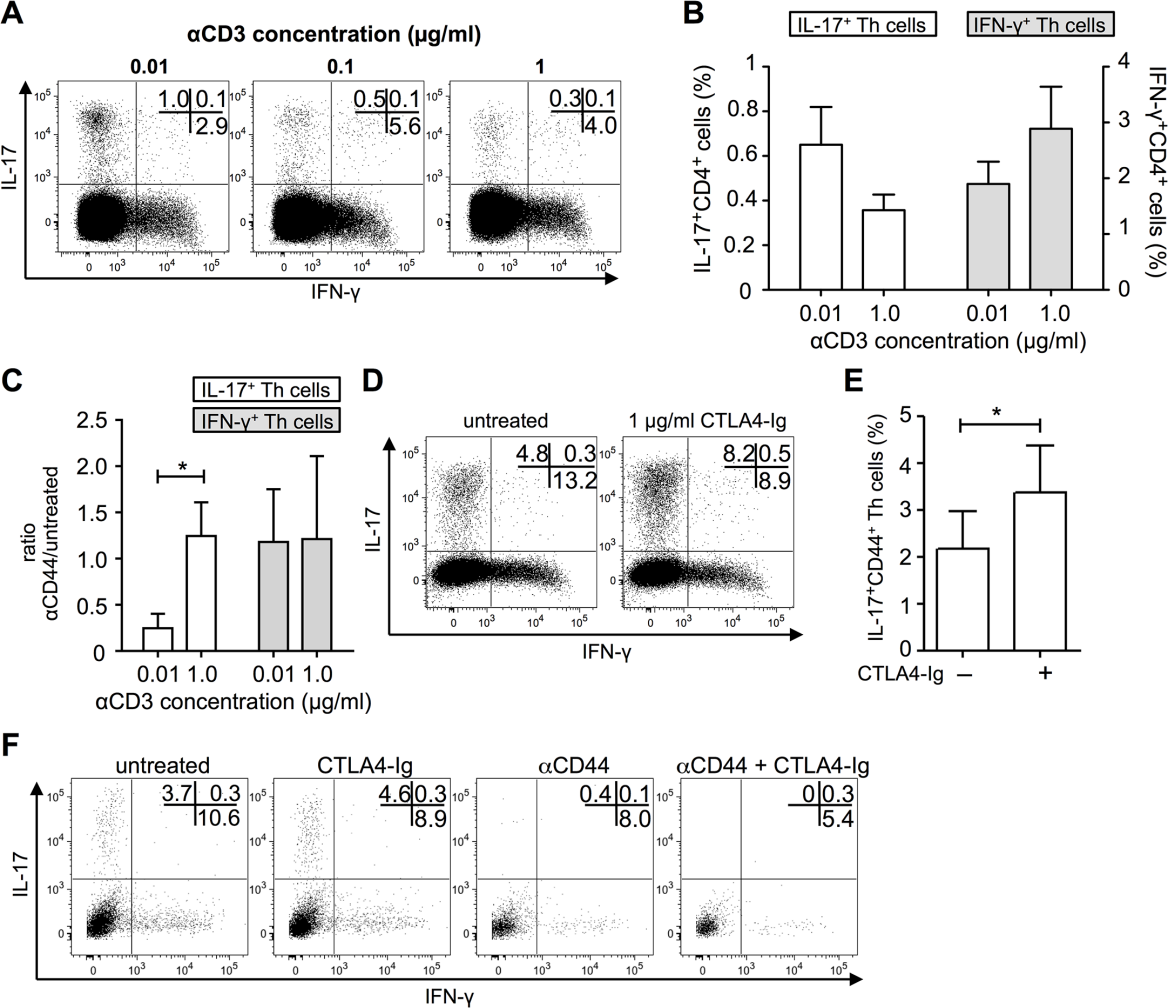
Low TCR and CD28 stimulation supports T_H_17 development. (A) IL-17 and IFN-γ production of CD4^**+**^ T cells stimulated with different amounts of αCD3. Percentage of positive cells is indicated in the dot plot. (B) Depicted is the IL-17 and IFN-γ production of CD4^**+**^ T cells stimulated with 0.01 or 1 μg/ml αCD3 (n = 6, *M* ± SEM, Wilcoxon rank sum test, between IL-17^**+**^CD4^**+**^ cells: not significant, between IFN-γ^**+**^CD4^**+**^ cells: *p* = 0.06). (C) Ratio of αCD44-treated and untreated IL-17^**+**^CD4^**+**^ or IFN-γ^**+**^CD4^**+**^ T cells stimulated with 0.01 or 1 μg/ml αCD3 (n = 3, *M* ± SEM, paired t test, between IL-17^**+**^CD4^**+**^ cells: *p ≤ 0.05, between IFN-γ^**+**^CD4^**+**^ cells: not significant). (D and E) Magnetically sorted CD4^**+**^ T cells were stimulated four days with allogeneic BMDCs. Co-cultures were left untreated or 1 μg/ml CTLA-4-Ig was added. Percentage of IL-17^**+**^CD4^**+**^ T cells of differently treated co-cultures is shown as a representative dot plot (D) and in a bar chart (E: n = 7, *M* ± SEM; Wilcoxon rank sum test, *p ≤ 0.05). (F) Magnetically sorted CD4^**+**^ T cells were stimulated four days with allogeneic BMDCs. Co-cultures were left untreated or were treated with 1 μg/ml CTLA-4-Ig or 10 μg/ml αCD44 or both. IL-17 and IFN-γ production of CD4^**+**^ T cells of the differently treated co-cultures is shown as a representative dot plot out of two independent experiments.

Föger *et al*. published that CD44 could act as co-stimulatory molecule and enhance TCR signaling under weak TCR stimulation conditions. Under these conditions it is able (at least in part) to replace CD28-signalling [12]. This was achieved by increasing the density of LCK at the immunological synapse, which is associated with CD44 [12, 13, 29, 30]. As we observed IL-17 producers being present in the CD44^+++^CD4^+^ population, we hypothesized that CD44 may act in IL-17 producing Th cells as a co-stimulatory molecule. To test whether this is true for our system, we first examined, whether weak stimulation conditions would favor IL-17 development. Therefore, we stimulated CD4^+^ T cells polyclonally with different amounts of αCD3 in the presence of syngeneic splenocytes. An allogeneic model would not be appropriate since the real antigen dose cannot be determined. As shown in Fig 5A and 5B, low TCR-stimulation favored development of IL-17^+^CD4^+^ T cells over generation of IFN-γ^+^CD4^+^ T cells. Although fewer cytokine-producing cells developed, in cultures with naïve-sorted CD4^+^ T cells IFN-γ production was also more affected upon reduced stimulation strength as compared to IL-17 production (S5 File). Importantly, CD44 blockade affected the development of IL-17^+^CD4^+^ T cells under low TCR-stimulation conditions but not under high TCR-stimulation (Fig 5C). Thus, high CD44 expression delivers crucial co-stimulatory signals to induce IL-17 production under low TCR signaling conditions, where additional co-signals are most critical. Furthermore, blockade of CD28 co-stimulation using the fusion protein CTLA-4-Ig, we wondered whether this also increased the frequency of IL-17-producing CD4^+^ T cells. Indeed, the frequency of IL-17-producers within activated CD4^+^ T cells increased after CTLA4-Ig treatment (Fig 5D and 5E). But here again, blockade of CD44 during CTLA4-Ig treatment reduced development of IL-17^+^CD4^+^ T cells (Fig 5F).

### High CD44 expression precedes formation of IL-17 producing T helper cells

**Fig 6:**
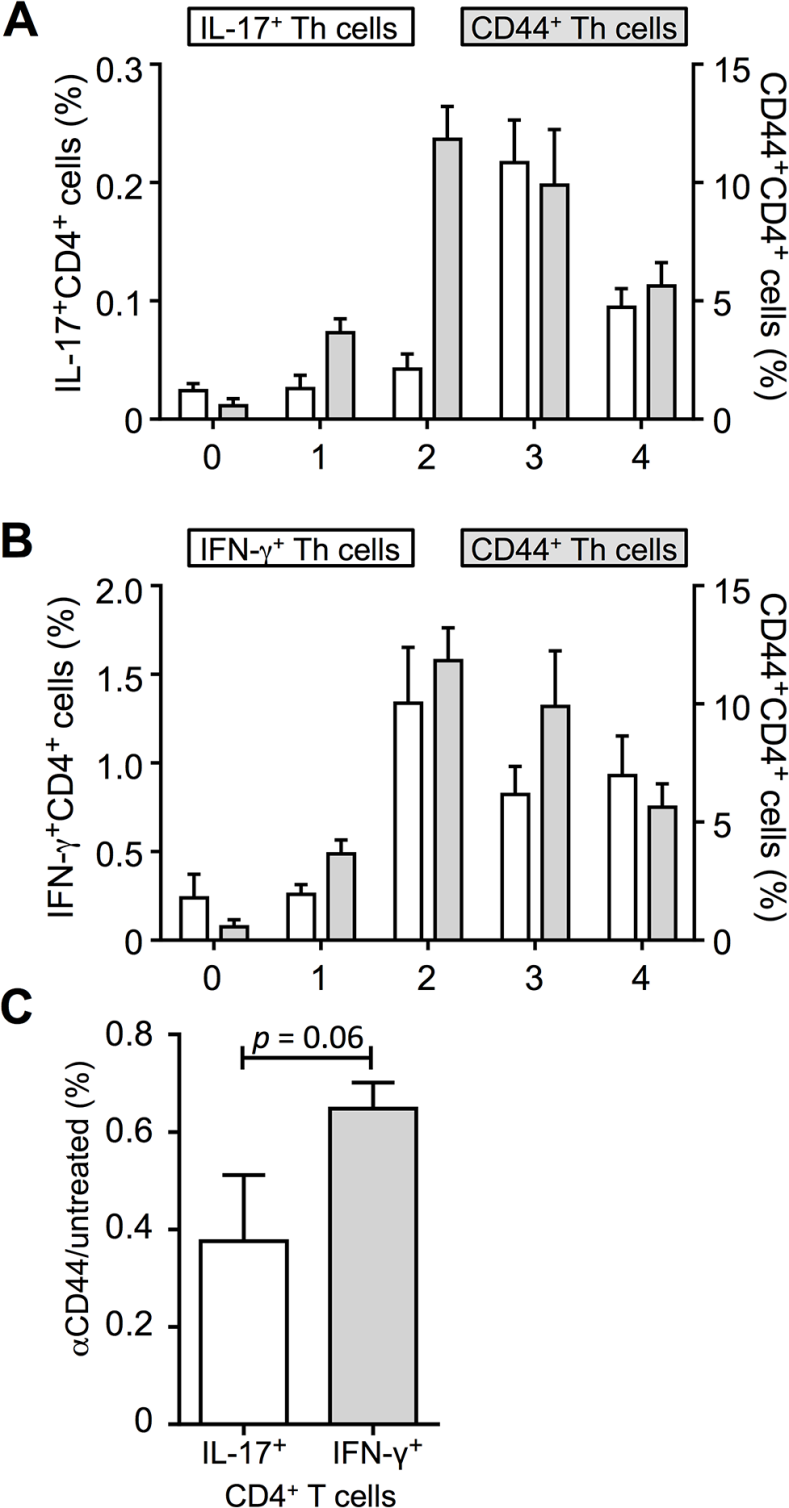
(A) IL-17 production (white bars, left Y-axis) and CD44 expression (grey bars, right Y-axis) of naïve-sorted CD4+ T cells before, one, two, three and four days after allogeneic stimulation with BMDCs (n = 4, ± SEM). (B) IFN-γ production (white bars, left Y-axis) and CD44 expression (grey bars, right Y-axis) of naïve-sorted CD4^**+**^ T cells before, one, two, three and four days after allogeneic stimulation with BMDCs (n = 4, ± SEM). (C) Magnetically sorted CD4^**+**^ T cells were stimulated four days with allogeneic BMDCs. Co-cultures were left untreated or were treated with 10 μg/ml αCD44 directly before PMA/ionomycin restimulation (n = 5, *M* ± SEM, Wilcoxon rank sum test, *p* = 0.06).

We hypothesize that a high CD44 led to an increased development of IL-17^+^ Th cells. To further address this question, we examined the kinetics of CD44 and IL-17 expression in naïve-sorted CD4^+^ T cells prior and after allogeneic stimulation. As it is shown in Fig 6A, CD44 expression peaked on day two, while most IL-17^+^CD4^+^ T cells were only measurable one day later, on day three. A decline in the CD44 expression (day three and four) was followed by a reduction of IL-17^+^CD4^+^ T cells (day four). In contrast, the IFN-γ production peaked at the same time as CD44 expression (Fig 6B).

Interestingly, by adding a αCD44 antibody at the same time as the re-stimulating agents PMA and ionomycin, we observed a reduction of IL-17^+^ and IFN-γ^+^ T cells as compared to samples without αCD44-treatment (Fig 6C). But again, IL-17 production was more affected than IFN-γ production. Indeed, these results indicate that high CD44 expression is important for inducing IL-17 production not so much for Th17 differentiation.

## Discussion

Our data show that co-culturing CD4^+^ T cells with allogeneic BMDCs resulted in the generation of three distinguishable populations regarding their CD44 expression: CD44^+^, CD44^++^ and CD44^+++^. Surprisingly, *in vitro* and *in vivo* generated allo-reactive IL-17 producing T helper cells were mainly found within the population with the highest CD44 expression, while IFN-γ producing T helper cells were CD44^++^. This profile was further enhanced under Th cell-polarizing conditions and neutralization of CD44 decreased Th17 rather than Th1 cells in the co-culture. Importantly, the difference in CD44 expression of IL-17 and IFN-γ producers was not due to previously formed memory T cells, as naïve-sorted CD4^+^ T cells also displayed this phenotype. CD45RB expression, is known to become down-regulated upon T cell activation [28] and was also reduced on IL-17 producing Th cells as compared to those producing IFN-γ supported our hypothesis, that Th17 cells have a higher cellular activation status. In line with this, we found the CD44^+++^CD4^+^ T cells to have the highest level of ZAP-70 phosphorylation. Under weak stimulation conditions CD44 can act as co-stimulatory molecule [12]. Under exactly these conditions we found a preferential development of IL-17producing Th cells. Thus, by enhancing the intracellular signalling cascade high CD44 expression can enable IL-17-producing Th cells to overcome suboptimal activation conditions.

Because CD45 is expressed in several isoforms [31], down-regulation of CD45RB does not mean an overall reduced expression of CD45 is reduced. But supporting its negative role during T cell activation CD45 is excluded from the central interaction zone of T cells and APCs because of its large extracellular part [32], which is not the case for LCK. Moreover, it has been shown that in CD45^−^ cells, a higher content of LCK is associated with CD44 in lipid rafts [33]. Thus, reduced CD45 expression supported higher cell activation status and was also directly linked to a CD44-mediated co-stimulation.

We also observed higher total ZAP-70 expression and phosphorylation levels within the CD44^+++^ population and importantly blocking CD44 reduced only the ZAP-70 phosphorylation of CD44^+++^ cells. Phosphorylation of ZAP-70 is one of the first events following T cell stimulation and downstream of LCK activation. Because many publications showed an association of LCK with CD44 [11–14], higher ZAP-70 phosphorylation in CD44^+++^CD4^+^ T cells could be attributed to a CD44-mediated co-stimulation. Since ZAP-70 acts downstream of LCK, higher CD44 surface levels are likely to increase the levels of LCK at the immunological synapse which in turn strengthened the intracellular signalling. Interestingly, we also measured higher total ZAP-70 levels in CD44^+++^ T cells as compared to subpopulations with intermediate or low CD44 expression. High ZAP-70 expression is observed in CD8^+^ T cell-precursors during thymic development. Saini *et al*. suggested that high ZAP-70 expression intracellularly amplifies a weak signal from a MHC I restricted ligand. Moreover, their data suggest that ZAP-70 expression is regulated by TCR signalling pathways, which means that ZAP-70 expression is regulated by a positive feedback loop [34]. Similar processes could be true for mature CD4^+^CD44^+++^ T cells.

CD44 molecules on APCs and T cells can interact using hyaluronan as a bridge [35]. Dendritic cells have been shown to actively synthesize hyaluronan [36] and thereby to contribute to this interaction. But conflicting data exist whether CD44 expression on dendritic cells or T cells influences T cell stimulation [37, 38]. Another possibility how CD44 could enrich LCK density at the immunological synapse is by a passive recruitment of lipid rafts during TCR-stimulation. LCK-CD44 association preferentially occurs in lipid rafts [13] and enrichment of lipid rafts could be induced upon TCR-triggering [29, 39]. Interestingly, our studies show that blocking during re-stimulation with PMA and ionomycin also blocked IL-17 secretion. These results indeed indicate that high CD44 expression is important for inducing IL-17 production not so much for Th17 differentiation. Th cell differentiation depends on different conditions, such as cytokine milieu, nature and dose of the antigen [17, 18, 40, 41]. Some publications showed an influence of the strength of T cell activation can influence the Th cell differentiation [41–43]. This can be mediated by different mechanisms such as co-stimulation, antigen dose, affinity and length of stimulation [26, 41]. Purvis *et al*. supported our observation that reduced αCD3-signalling promote Th17 development which does not result in reduced NFAT translocation to the nucleus [26]. This confirmed our data that weak TCR stimulation did not necessarily result in decreased cell activation.

However, it is controversially discussed whether low TCR-stimulation results in enhanced Th17-differenciation [26, 44, 45] as some publications showed an association of Th1 differentiation with enhanced intracellular signalling. Deficiency of the induced T cell kinase led to a reduced IL-17 levels [45] and raftlin-KO mice also showed decreased IL-17 production. Raftlin is a protein located in lipid rafts and controls TCR signaling strength. Raftlin containing lipid rafts also contain LCK and might also control the LCK density [46].

Influence of different co-stimulatory molecules on Th17 cell differentiation has been described, such as CD28 and ICOS [19]. Alike for αCD3 stimulation, controversial data exist whether CD28 and ICOS support or inhibit Th17 differentiation [26, 27, 47, 48]. The data shown here support rather the view that a reduced CD28-stimulation promote Th17 differentiation [27] which is in line with some *in vivo* data where the administration of CTLA-4-Ig worsened the progress of EAE [49]. Moreover, a phase III trial in kidney transplantation showed that Belatacept dose-dependently increased the acute rejection rate [50]. When the T cell phenotype within protocol biopsies of renal transplant recipients was analyzed, it was noticed that some samples from Belatacept-treated patients expressed higher levels of IL-17 compared to samples from patients receiving a calcineurin inhibitor-based treatment protocol [51]. Interestingly, this could not be observed for intragraft IFN-γ expression. Furthermore, in a rheumatoid arthritis synovium SCID mouse model it was shown that only neutralization of IL-17 but not application of CTLA-4-Ig has a therapeutic effect on CD3-rich samples [30]. CTLA-4-Ig inhibited the IL-2 secretion [49] and IL-2 in turn inhibits Th17-differentiation [52]. Therefore decreased IL-2 levels could in some cases enhance Th17 immune response.

We hypothesize that a high CD44 expression modulates cell activation, which facilitates development of IL-17 producing T cells during low TCR stimulation. We could show, that the formation of IL-17 producing T helper cells is delayed in time as compared to CD44 up-regulation following T cell activation. In contrast development of IFN-γ production showed the same time course as CD44 up-regulation. This delay in time and the difference to IFN-γ supports this hypothesis because it shows that IL-17 production is only induced when the T helper cells have acquired a high CD44 expression.

Although weak stimulatory conditions might not always be a prerequisite for Th17 stimulation, our data showed that Th17 cells developing under these conditions might have an advantage due to enhanced CD44 surface expression. Th17 cells express homing receptors for the skin and mucosa [53]. Especially the skin contains a huge amount of hyaluronan [54]. *In vivo* Th17 cells were found at early stages of an immune response, which at later phases shifts to a Th1 response [40, 55]. This suggests a potential advantage of Th17 cells during early immune responses. In this context CD44 crosslinking can act as a co-stimulus and enhance T cell signalling. Another way how CD44 might enhance *in vivo* responses of Th17 cells is via its function as an adhesion protein. CD44 mediates adhesion to the extracellular matrix and extravasation of lymphocytes to inflamed tissues [56]. Also in this context high CD44 expression could be beneficial during early responses.

Taken together our data show, that allo-reactive Th17 cells were characterized by a high CD44 expression, which modulated the cell activation status. This enabled Th17 cells to respond to low stimulation conditions.

## Supporting Information

S1 File Scheme showing the gating strategy. (TIFF)

S2 File Different IL-17^+^ and IFN-γ^+^ CD4^+^ T cell generations can be distinguished according to their CD44 surface expression. (TIFF)

S3 File CD44^+++^CD4^+^ T cells have higher phospho-ZAP-70 levels. (TIFF)

S4 File CD44^+++^CD4^+^ T cells have higher ZAP-70 levels. (TIFF)

S5 File IFN-γ secretion is more affected by a low TCR stimulation than IL-17 secretion. (TIFF)
