# Correction: Differences in CD44 Surface Expression Levels and Function Discriminates IL-17 and IFN-γ Producing Helper T Cells

**DOI:** 10.1371/journal.pone.0143986

**Published:** 2015-11-24

**Authors:** Julia Schumann, Katarina Stanko, Ulrike Schliesser, Christine Appelt, Birgit Sawitzki

The following information is missing from the Funding section: e:Kid from the Federal Ministry of Education and Research (BMBF). The correct funding information is as follows: The work was supported by the grants Transregio 52 from the German Research Foundation (DFG) and e:Kid from the Federal Ministry of Education and Research (BMBF) to B.S. The funders had no role in study design, data collection and analysis, decision to publish, or preparation of the manuscript.
