# Supplementary figures and images for: Differences in CD44 Surface Expression Levels and Function Discriminates IL-17 and IFN-γ Producing Helper T Cells
Source: PLoS One. 2015 Jul 14;10(7):e0132479. doi: 10.1371/journal.pone.0132479 (PMC4501817; doi:10.1371/journal.pone.0132479)

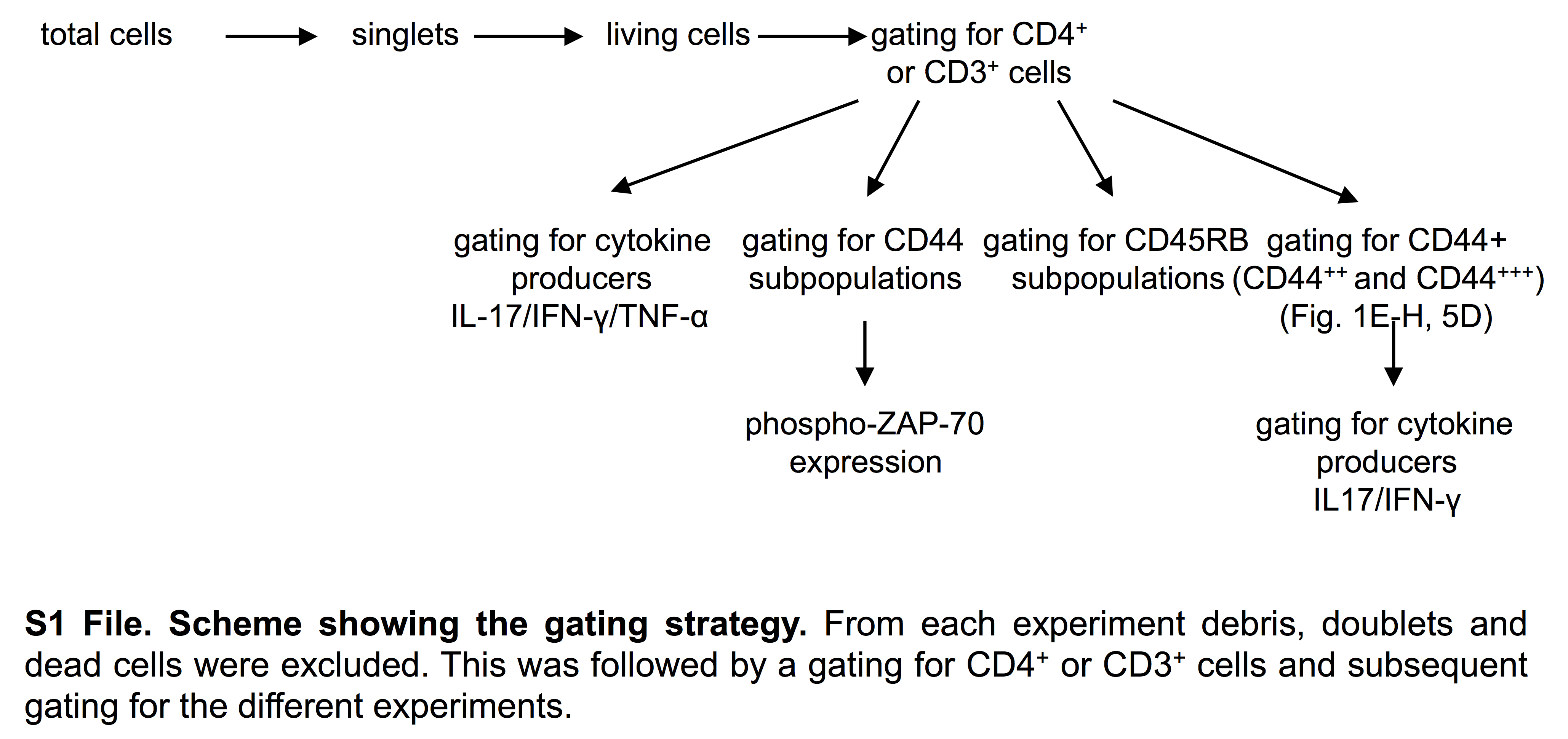

Supplement: S1 File — (TIFF) [file pone.0132479.s001.tiff]

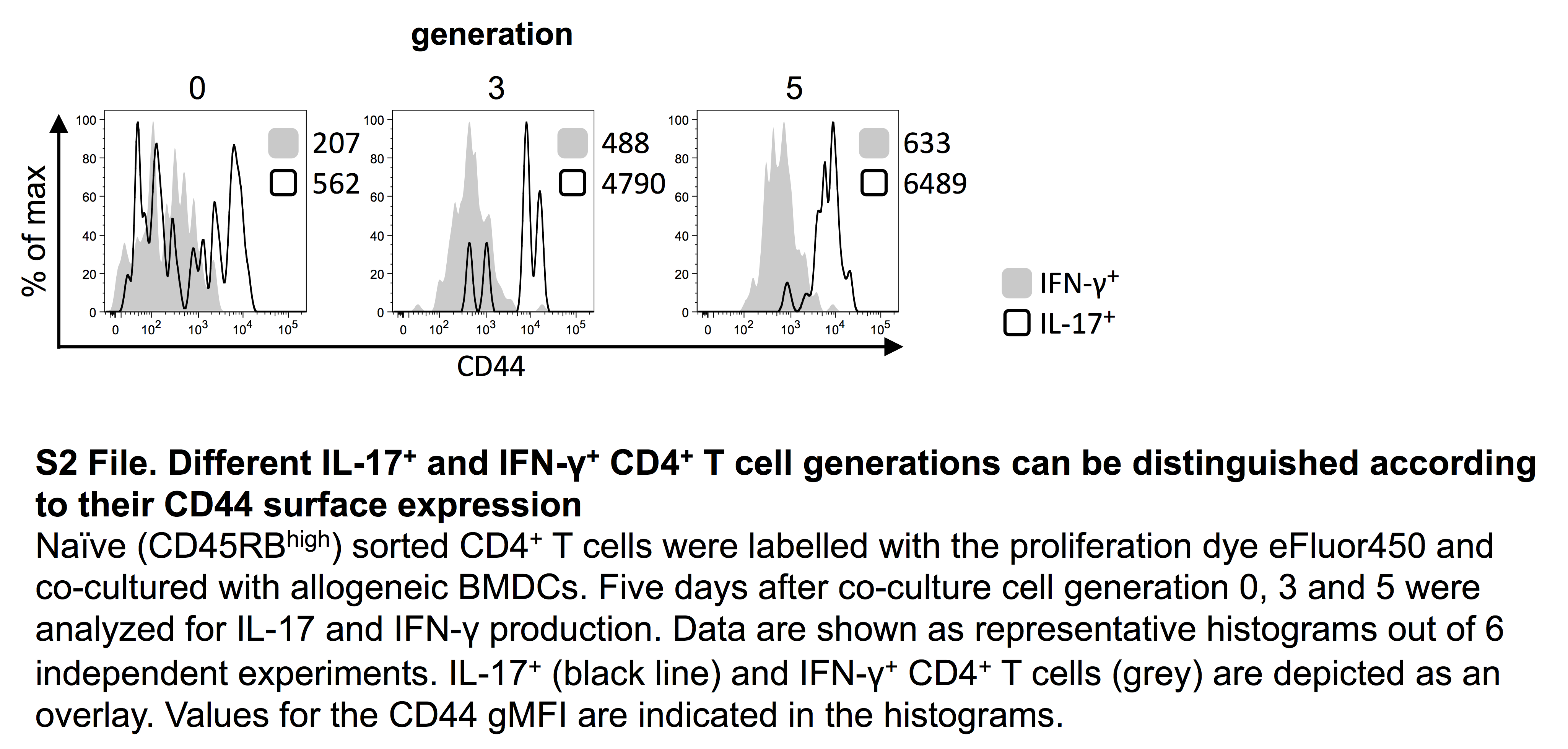

Supplement: S2 File — (TIFF) [file pone.0132479.s002.tiff]

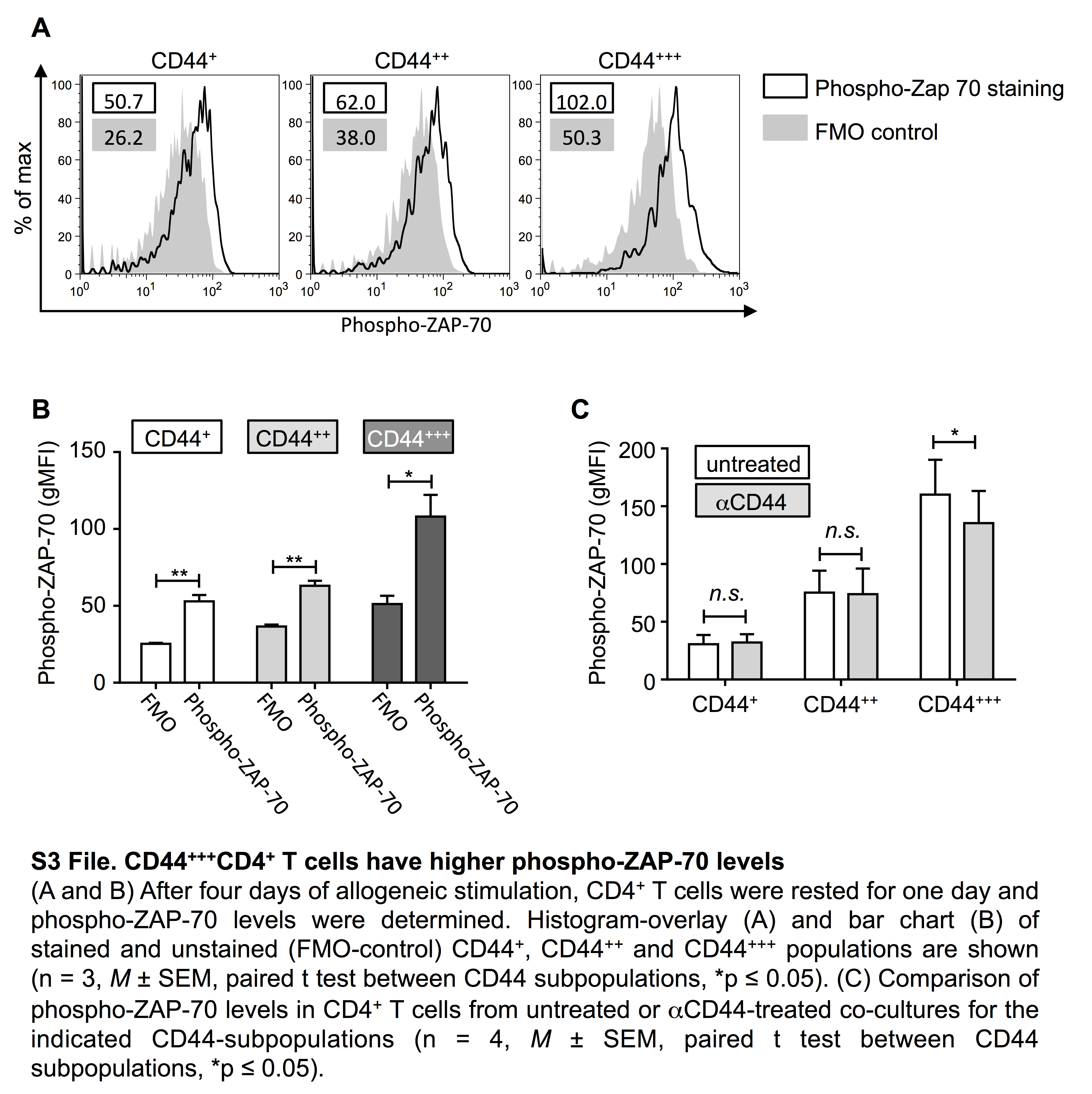

Supplement: S3 File — (TIFF) [file pone.0132479.s003.tiff]

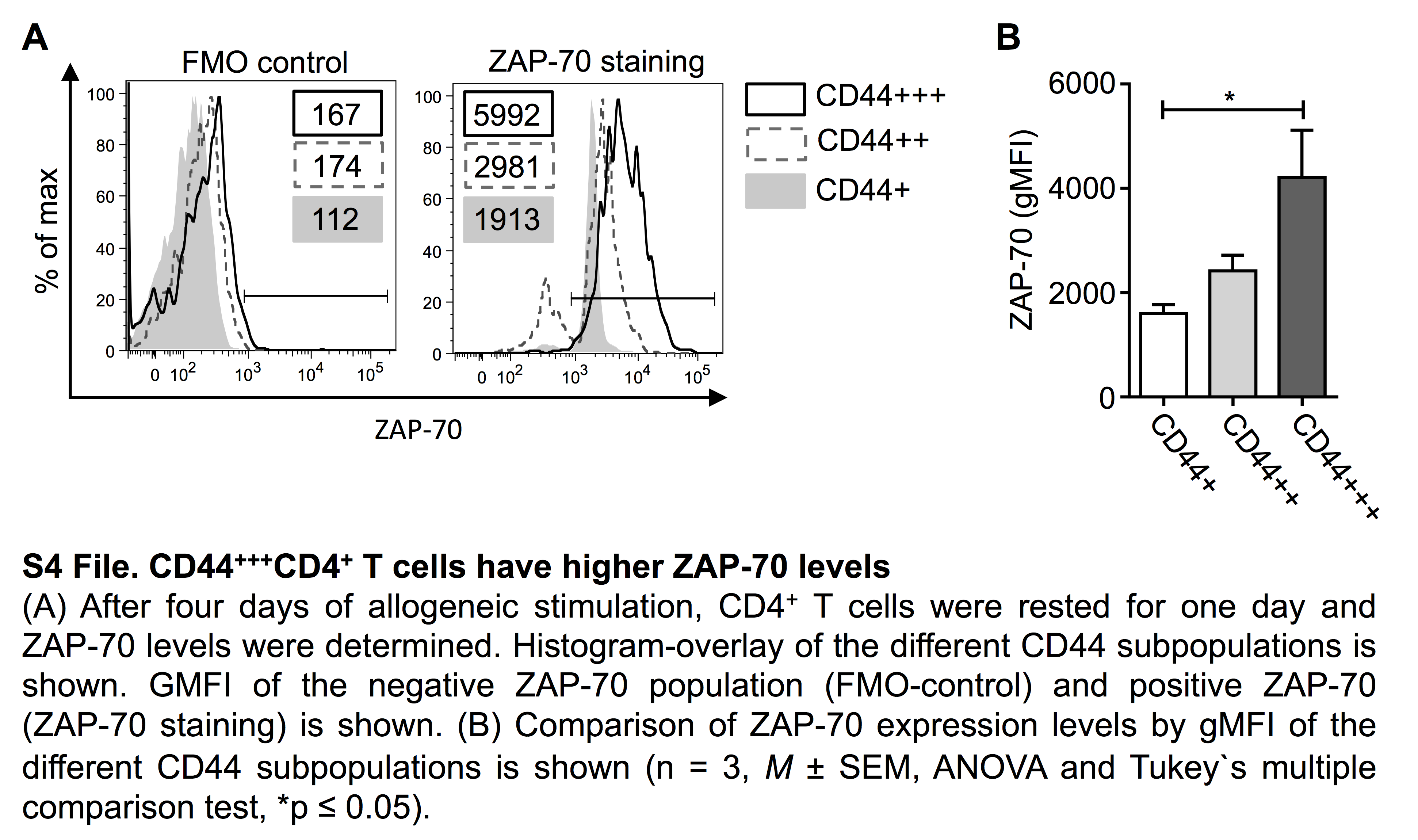

Supplement: S4 File — (TIFF) [file pone.0132479.s004.tiff]

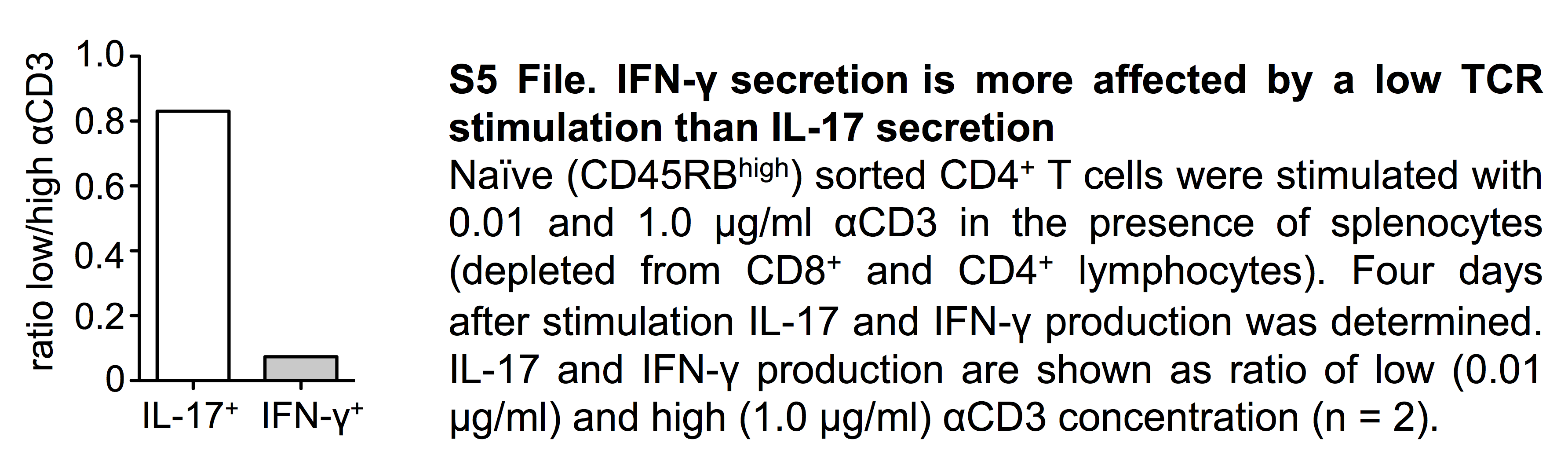

Supplement: S5 File — (TIFF) [file pone.0132479.s005.tiff]
